# Rabbit hemorrhagic disease virus 2, 2010–2023: a review of global detections and affected species

**DOI:** 10.1177/10406387241260281

**Published:** 2024-06-19

**Authors:** Javier Asin, Carlos Calvete, Francisco A. Uzal, Beate M. Crossley, Margarida Dias Duarte, Eileen E. Henderson, Fábio Abade dos Santos

**Affiliations:** California Animal Health and Food Safety Laboratory, University of California–Davis, San Bernardino, CA, USA; Animal Science Department, Agri-Food Research and Technology Centre of Aragon (CITA), Agri-Food Institute of Aragón (IA2), Zaragoza, Spain; California Animal Health and Food Safety Laboratory, University of California–Davis, San Bernardino, CA, USA; Davis, CA, USA; National Institute for Agrarian and Veterinary Research (INIAV), Oeiras, Portugal; California Animal Health and Food Safety Laboratory, University of California–Davis, San Bernardino, CA, USA; National Institute for Agrarian and Veterinary Research (INIAV), Oeiras, Portugal; Faculty of Veterinary Medicine, Lusofona University, Lisboa, Portugal

**Keywords:** cottontails, GI.2, hares, jackrabbits, *Lagovirus europaeus*, rabbits, rabbit hemorrhagic disease, rabbit hemorrhagic disease virus 2, *Sylvilagus*

## Abstract

Rabbit hemorrhagic disease virus 2/genotype GI.2 (RHDV2/GI.2; *Caliciviridae*, *Lagovirus*) causes a highly contagious disease with hepatic necrosis and disseminated intravascular coagulation in several *Leporidae* species. RHDV2 was first detected in European rabbits (*Oryctolagus cuniculus*) in France in 2010 and has since spread widely. We gather here data on viral detections reported in various countries and affected species, and discuss pathology, genetic differences, and novel diagnostic aspects. RHDV2 has been detected almost globally, with cases reported in Europe, Africa, Oceania, Asia, and North America as of 2023. Since 2020, large scale outbreaks have occurred in the United States and Mexico and, at the same time, cases have been reported for the first time in previously unaffected countries, such as China, Japan, Singapore, and South Africa, among others. Detections have been notified in domestic and wild European rabbits, hares and jackrabbits (*Lepus* spp.), several species of cottontail and brush rabbits (*Sylvilagus* spp.), pygmy rabbits (*Brachylagus idahoensis*), and red rock rabbits (*Pronolagus* spp.). RHDV2 has also been detected in a few non-lagomorph species. Detection of RHDV2 causing RHD in *Sylvilagus* spp. and *Leporidae* species other than those in the genera *Oryctolagus* and *Lepus* is very novel. The global spread of this fast-evolving RNA virus into previously unexploited geographic areas increases the likelihood of host range expansion as new species are exposed; animals may also be infected by nonpathogenic caliciviruses that are disseminated by almost all species, and with which genetic recombination may occur.

## The order *Lagomorpha*

**Figure 1.**
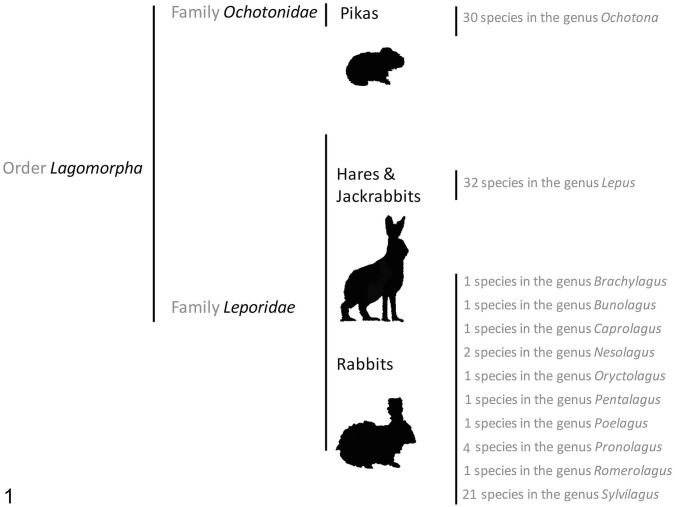
The order *Lagomorpha* with genera in families *Ochotonidae* (pikas) and *Leporidae* (leporids). Adapted from reference 2 and updated per the International Union for Conservation of Nature–Species Survival Commission, Lagomorph Specialist Group.^91^

The order *Lagomorpha* (Fig. 1) has a long evolutionary history that can be traced back to 55 million years ago in Mongolia.^15^ It comprises 2 families: *Ochotonidae* (pikas) and *Leporidae* (rabbits, hares and jackrabbits). The *Leporidae* family is the largest, with 66 species classified into 11 different genera.^2,91^ Rabbits are small lagomorphs divided into 10 genera; hares and jackrabbits are usually larger and are grouped in a single genus, *Lepus*. Several species within the *Leporidae* family face conservation challenges and, according to the International Union for Conservation of Nature, some species are categorized as vulnerable, threatened, or critically endangered.^2,91^

*Leporidae* species can be found on all continents except Antarctica. *Lepus* is the most diverse genus and has a global distribution.^11,62^ The European rabbit (*Oryctolagus cuniculus*) is the single species in the genus *Oryctolagus* and arguably the best-known lagomorph; it is native to the Iberian Peninsula and southern France but wild European rabbits have been introduced into many other areas of the world, including the rest of Europe, certain parts of South America, most of Oceania, and numerous islands.^11^ Domestic European rabbits are often kept as pets, and used for research and for meat and fur production, among other purposes. Feral populations of domestic European rabbits have proliferated in the wild and are found in various parts of the world. The genus *Sylvilagus* is native to the Americas and is widely distributed throughout North and South America, although some species are restricted to certain regions.^110,132^ There is also an established population of eastern cottontail (*Sylvilagus floridanus*) in Italy that was introduced for hunting purposes in the 1960s.^58^

Leporids play a crucial role in many ecosystems. They are primary consumers that convert plant protein into animal protein of different weight ranges depending on the species, providing an ideal biomass intake for predators of various sizes, including genets, wild felids and canids, and eagles.^56,161,167,173^ The decline of native *Leporidae* species due to diseases such as myxomatosis or rabbit hemorrhagic disease (RHD) can lead to significant changes in the structure and function of natural ecosystems, directly impacting intermediate and top predators, and indirectly affecting other species within interconnected food webs. The wild European rabbit is a particularly well-studied *Leporidae* species and serves as a crucial food source for >40 terrestrial and aerial carnivore species in the Iberian Peninsula. The Iberian lynx and the Spanish imperial eagle, which include wild rabbits in ~90% of their diet, are among the carnivores that rely on this species.^2,57,126^

## Overview of lagoviruses

The genus *Lagovirus* belongs to the family *Caliciviridae* in the order *Picornavirales*. Until 2023, the genus included 2 species: European brown hare syndrome virus (EBHSV; GD/FR/1989) and rabbit hemorrhagic disease virus (RHDV; GH/DE/1988; EBHSV and RHDV are antigenically and genetically similar [53–70% nucleotide homology in the *vp60* gene]).^107,133^ The primary hosts of EBHSV and RHDV are the European brown hare (*Lepus europaeus*) and the European rabbit, respectively. RHD is listed by the World Organisation for Animal Health (WOAH) as a notifiable disease of terrestrial animals due to its transmissibility and significant impact on socioeconomic factors and the international trade of animals and animal products.^199^

**Figure 2.**
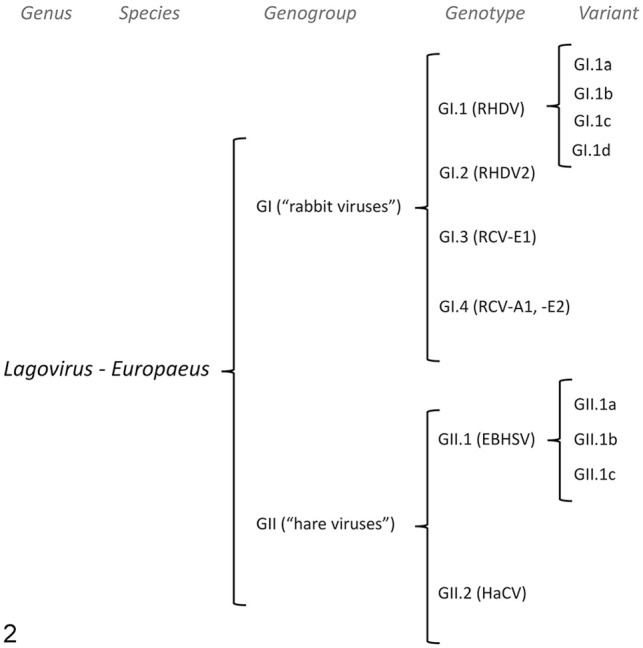
Organization of the *Lagovirus* genus based on *vp60* sequences.^107^ Genotypes are established based on genetic differences of >15%. Variants are established based on genetic differences of >6%. EBHSV = European brown hare syndrome virus; HaCV = hare calicivirus (nonpathogenic); RCV = rabbit calicivirus (nonpathogenic); RHDV = rabbit hemorrhagic disease virus.

In 2017, several authors proposed a new classification based on phylogeny (Fig. 2), specifically by aligning *vp60* gene sequences to determine major lineages.^107^ This classification is widely used in the scientific literature about RHD (e.g.,^6,160^). In 2023, the International Committee on Taxonomy of Viruses (ICTV) officially adopted this classification and merged EBHSV and RHDV into *Lagovirus europaeus*,^90^ which is divided into 2 genogroups: GI, which includes rabbit viruses (RHDV and related nonpathogenic rabbit caliciviruses [RCVs]); and GII, which includes hare viruses (EBHSV and related nonpathogenic hare caliciviruses [HaCVs]). These genogroups are further subdivided into different genotypes, each with a genetic distance of >15% from the others. The GI.1 genotype includes the first identified strains of RHDV (i.e., “classic” RHDV); the GI.2 genotype represents more recent strains classified as RHDV2, which was first detected in France in 2010, and has spread worldwide, gradually replacing previously circulating GI.1 viruses.^107,151^ The GI.3 and GI.4 genotypes include nonpathogenic RCVs, which are known to circulate silently in rabbit populations. Finally, within some genotypes, there are further subdivisions into different variants based on genetic distances >6%.^107^ Therefore, 2 different viruses can cause RHD: RHDV (also known as *Lagovirus europaeus* GI.1 or simply GI.1) and RHDV2 (also known as RHDVb, *Lagovirus europaeus* GI.2, or simply GI.2).

Lagoviruses are single-stranded, positive-sense RNA viruses with an icosahedral capsid, a spherical shape, and no envelope; the viral capsid is composed of 90 arch-like dimers of the capsid protein VP60 and has 32 cup-shaped depressions reminiscent of a calyx, which gives the viral family the name *Caliciviridae*.^46,90,136,140^ The major capsid protein VP1/VP60 forms the structure of the virion; the minor structural protein VP2/VP10 provides stability following encapsidation of the viral RNA.^139,140,170^ The viral RNA is covalently linked to the viral genome–linked protein (VpG), which is essential for replication.^115,123^ The viral genome is ~7.4 kb long and contains an additional ~2.2 kb subgenomic RNA that binds to it. The genomic RNA is divided into 2 regions: ORF1, which encodes a polyprotein cleaved into nonstructural proteins (p16, p23, a 2C-like helicase, p29, the VpG, a 3C-like protease, and an RNA-dependent RNA polymerase [RdRp]) and the major structural capsid protein VP60; and ORF2, which encodes the minor structural protein VP10.^136,174^ The VP60 protein is the major viral antigen and has a surface loop called L1 in the P2 subdomain. This loop varies among strains and contains epitopes that induce the production of neutralizing antibodies. Cross-protective immunity among genogroups and genotypes is limited, suggesting that new viruses are likely to emerge within those levels.^31,39,40,50,98,154^

As with other members of the *Caliciviridae* family, inter-genomic recombination between different strains is a very important evolutionary feature of lagoviruses.^117^ It usually occurs at the junction between the nonstructural and structural genes (*RdRp*-*vp60*), and various combinations involving both pathogenic and nonpathogenic lagoviruses have been described.^6^ Recombinants are named with the genotype of the nonstructural donor with a “P” (standing for “polymerase”), followed by the structural donor.^107^ For example, a GI.3P-GI.2 virus would be a RHDV2 recombinant virus between the nonstructural genes of a nonpathogenic RCV/GI.3 and the structural genes of a pathogenic RHDV2/GI.2. In addition, a less-common second recombination breakpoint at the *p16-p23* boundary has been described, allowing for the occurrence of triple recombinants.^155^ In this review, we will use RHDV2 generically to refer to viruses that have been identified as having GI.2 structural (capsid) protein genes, and RHDV to viruses that have been identified as having GI.1 structural (capsid) protein genes, regardless of the complete genotype; when referring to the genotype of specific recombinants, we will use the aforementioned nomenclature (i.e., GX.XP-GI.2 for RHDV2 recombinants).

Typical clinical signs of RHD are the result of severe hepatic failure and dysregulation of the coagulation system. The main differences between RHDV and RHDV2 are that the latter has a much wider host range within the *Leporidae* family, including several *Lepus* and *Sylvilagus* species, and most likely others, in addition to *Oryctolagus*, and is capable of infecting and causing disease in very young individuals.^18,37,51,142^ Other potential differences include a slightly longer incubation period for RHD caused by RHDV2, more variable and possibly lower apparent mortality for RHDV2, and possibly a higher proportion of rabbits with subacute or chronic presentations with RHDV2. However, highly virulent RHDV2 strains with mortality rates similar to those of RHDV have been described.^38^ According to the WOAH guidelines, a method that is able to differentiate among RHDV2, RHDV, and other lagoviruses must be used to confirm a diagnosis of RHD caused by RHDV2, with RT-PCR or RT-qPCR on liver as the current method(s) of choice in most diagnostic laboratories.^198^ A comprehensive review of diagnostic tests is available elsewhere.^8^

## Objectives and methodology

In this literature review, we gathered data on RHDV2 detections reported worldwide, focusing on reports in different lagomorph species, pathology and genetic differences, and diagnostic advances made in the context of the global spread of this virus in each geographic location. We searched the scientific literature (PubMed, Google Scholar, Web of Science, and Scopus) and the websites of official international, national, and regional animal health regulatory agencies and authorities (e.g., WOAH, U.S. Department of Agriculture–Animal and Plant Health Inspection Service [USDA-APHIS], or as indicated below).

## Global detections

**Figure 3.**
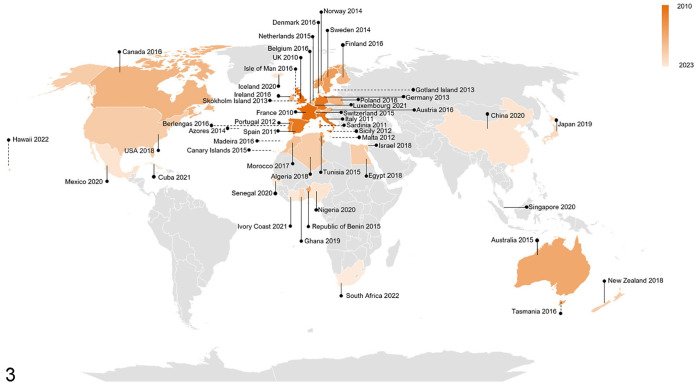
World map of rabbit hemorrhagic disease virus 2 detections reported between 2010 and 2023 in different countries (solid lines) and islands (dashed lines). Countries and islands with reported detections are in orange; earlier detections are darker, and the latest detections are lighter. Countries with no reported detections are in gray. References: Algeria,^152^ Australia,^76^ Austria,^19^ Azores,^60^ Belgium,^84^ Benin,^176^ Berlengas,^3^ Canada,^13^ Canary Islands,^121^ China,^179^ Cuba,^181^ Denmark,^182^ Egypt,^67^ Finland,^92^ France,^105^ Germany,^70^ Ghana,^12^ Gotland,^130^ Hawaii,^157^ Iceland,^183^ Ireland,^149^ Isle of Man,^149^ Israel,^184^ Italy,^186^ Ivory Coast (RHDV2 was most likely present at least since 2016^187^),^188^ Japan,^96^ Luxembourg (RHDV2 was most likely present at least since 2015–2017^120^),^189^ Madeira,^43^ Malta,^169^ Mexico,^190^ Morocco,^114^ Netherlands,^63^ New Zealand (RHDV2 was most likely present at least since 2017^124^),^191^ Nigeria,^55^ Norway,^192^ Poland,^69^ Portugal,^7^ Sardinia,^142^ Senegal,^193^ Sicily,^34^ Singapore,^163^ Skokholm,^156^ South Africa,^195^ Spain,^51^ Sweden,^130^ Switzerland,^9^ Tasmania,^116^ Tunisia,^144^ United Kingdom,^171^ United States.^196^ Map developed using Bing technology and adapted.

By October 2023, RHDV2 had been detected almost globally (Fig. 3). It is actively spreading to new territories and readers are encouraged to check the official reporting agencies’ websites for the latest information on reports of detection of this virus (e.g., https://wahis.woah.org/#/event-management).

### Europe

The first detections of RHDV2 occurred almost simultaneously in different parts of Europe at the beginning of the first decade of the 2000s, and the virus is thought to have emerged on that continent. The first reports of confirmed RHDV2 detections were from northwestern and central France in April–May 2010, with rapid spread in domestic and wild European rabbit populations.^103,105^ At the same time as RHDV2 was being detected in several countries in continental Europe, the authors of a 2015 study reported that the virus was also present in 2 locations in England since at least May 2010, based on detections in archived samples; these represented the first confirmed detections in the entire United Kingdom.^171^ Given the geographic distance between the detections and the low level of RHD surveillance in England at the time, the authors suggested that RHDV2 may have been circulating in Great Britain before that year.^171^ The virus continued to circulate in Great Britain and was also detected in Scotland and Wales in following years.^20,171^

RHDV2 was first detected in domestic and wild rabbits in the northeast of Spain in May 2011.^33,51^ However, mortality events associated with a disease compatible with RHD (although not confirmed by laboratory testing) were observed earlier that year in farmed rabbits vaccinated against RHDV in eastern Spain (regions of Catalonia and Valencia), suggesting that the incursion of RHDV2 into Spain may have occurred earlier.^73^ Almost simultaneously, in July 2011, RHDV2 was also detected in a rabbitry in northeastern Italy, and cases were also observed that year on the Italian island of Sardinia.^103,142,186^ Later, in 2012, reports of RHDV2 emerged on the Italian island of Sicily^34^ and possibly on Malta (A. Lavazza and L. Capucci, unpublished results mentioned in a 2017 study^169^).

RHDV2 was detected in wild rabbits from the north and south of mainland Portugal between November 2012 and February 2013,^7^ in wild rabbits from the Swedish island of Gotland in May 2013,^130^ and in domestic rabbits in Germany in 2013.^70^ Cases were detected in the uninhabited island of Skokholm, near the Welsh Coast, in 2013.^156,171^ Further analyses of wild rabbits revealed the circulation of RHDV2 in the Portuguese Azores islands in 2014, probably due to a previous virus introduction from southern Portugal.^59,60^ RHDV2 was detected in domestic rabbits in Norway in 2014.^192^

In 2015, RHDV2 was detected in domestic rabbits in Switzerland and the Netherlands.^9,63^ In 2016, detections were reported in domestic rabbits in Austria,^19^ Belgium,^84^ and Denmark,^182^ and in domestic, wild, and/or feral domestic rabbits in Finland,^92^ the Republic of Ireland,^149^ Poland,^69^ the Isle of Man,^149^ and the Portuguese archipelagos of Berlengas and Madeira.^3,43^ Phylogenetic analyses of the Madeira viruses again suggested a possible introduction from the south of mainland Portugal. Detections of RHDV2 were first reported to WOAH in domestic rabbits in Iceland and Luxembourg in 2020 and 2021,^183,189^ respectively; however, a 2018 study mentioned detections of RHDV2 in samples of domestic rabbits from Luxembourg collected between 2015 and 2017.^120^ RHDV2 was most likely detected in Croatia in 2023^180^; the latter country has not reported the virus type yet, but per the mentioned WOAH report, the detection was in a European brown hare, suggesting that it was RHDV2 rather than RHDV.

The broader host range of RHDV2 was first recognized in Europe when the virus was detected in association with disease in hares from several countries (see also section on *Lepus*). During the autumn and winter of 2011, an outbreak of a disease compatible with RHD affected a significant number of Cape hares (syn. Sardinian Cape hares; *Lepus capensis mediterraneus*) in Sardinia, Italy, and was confirmed to be caused by RHDV2.^142^ The following year, RHDV2-associated mortality was reported in Italian hares (syn. Corsican hares; *Lepus corsicanus*) and European brown hares, also in Italy, followed by detections in European brown hares in France in 2013 and Spain in 2014.^34,104,169^ Cases in mountain hares (*Lepus timidus*) were reported between 2016 and 2017 in Sweden and Germany.^25,129^ In 2017, the virus was detected in hares of unspecified species in the Netherlands.^64^ In 2018–2019, the virus was detected causing mortality in Irish hares (*Lepus timidus hibernicus*) in Ireland,^97^ European brown hares in England and Scotland,^21,148^ and, shortly thereafter, in Iberian hares (syn. Granada hares; *Lepus granatensis*) in Spain in 2020.^168^

In countries in which RHDV2 was detected, there was rapid viral spread, and this new virus quickly replaced the classic RHDV strains that had been circulating. The spread of RHDV2 and the replacement of RHDV strains was faster in areas in which wild rabbit populations were abundant and the competitive advantages of RHDV2 over RHDV were enhanced,^33,103^ so that RHDV strains were almost completely replaced in <2 y after emergence in countries such as France,^103^ Spain,^50^ and Portugal.^112^ However, in other countries, such as Germany and Poland, RHDV continued to co-circulate with RHDV2 for longer times.^87,160^

Shortly after its emergence, recombinant RHDV2 strains were detected in the Iberian Peninsula,^113^ France,^104^ and the Portuguese archipelagos of Azores and Madeira,^10,111^ infecting rabbits and also hares.^104,168^ Initially, most of the recombination events identified involved the junction between the genes encoding for VP60 (structural proteins) and RdRp (nonstructural proteins); RHDV2 (genotype GI.2) was the donor of the structural proteins, whereas the nonstructural proteins originated from either a pathogenic RHDV (genotype GI.1) or a nonpathogenic RCV (genotypes GI.3 or GI.4). RHDV2 recombinants encoding the nonstructural genes of EBHSV (genotype GII.1) were also reported to infect hares in Germany.^160^ Furthermore, a second recombination breakpoint was detected in Portugal at the junction of the 2 genes encoding the nonstructural proteins p16 and p23, allowing the existence of triple recombinants in which RHDV2 was the donor of the structural part, a nonpathogenic lagovirus was the parental donor of p16, and RHDV genotype GI.1b was the donor of the rest of the nonstructural part.^155^ The latter illustrates the high potential of lagoviruses to increase genetic diversity, leading to the emergence of new pathogenic strains. There is strong evidence that recombination contributed to the emergence of RHDV2 as a pathogenic virus in Europe, as phylogenetic and recombination analyses have shown that the first RHDV2 strains, previously considered as non-recombinants, arose from a recombination event between the GI.3 and GI.2 genotypes.^6^

Detection methods evolved rapidly in response to the emergence of RHDV2 in Europe. Although it was initially reported that the virus did not agglutinate human RBCs of type O or A,^51^ further studies demonstrated that RHDV2 efficiently agglutinated RBCs of type O at titers similar to those seen with most classic RHDVs, and hemagglutination (HA) was therefore often used to screen for RHDV2.^103^ However, because HA cannot be used to differentiate between different lagoviruses, after the emergence of RHDV2, viral detection was mainly performed by amplification and sequencing of the partial or complete gene encoding VP60 (e.g.,^50,103,105,130^). Subsequently, given the different antigenic profile of RHDV2, differentiation from RHDV could also be performed using anti-RHDV monoclonal antibodies and a sandwich ELISA.^103,142^ Nevertheless, procedures based on RT-qPCR targeting a specific nucleotide RNA region located within the *vp60* gene have been, and are by far, the most widely used assays for RHDV2 detection and viral load assessment due to their high specificity, sensitivity, and efficiency.^48,61^ Interestingly, RT-qPCR can detect RHDV2 RNA in commercial inactivated vaccines, but cannot detect vaccine RNA in rabbits vaccinated 15 d before, enabling the detection of circulating field strains in rabbitries following an RHD outbreak and subsequent vaccination.^42^ RT-qPCR was also useful to demonstrate the likely presence of persistent infections, carriers, and unnoticed virus circulation in rabbitries after an RHD outbreak.^29,41,122^ Similarly, a duplex RT-qPCR designed to detect RHDV (genotype GI.1b) and RHDV2 RNA in a single analysis was developed and used to monitor RHD outbreaks and interactions between the 2 different viral types in wild rabbit populations.^28,30,32^

Despite the undoubted usefulness of PCR-based methods, other less-sensitive detection tests have been developed in Europe, such as a sandwich ELISA for the detection of RHDV2 antigen in rabbit liver homogenates,^53^ which allowed the detection of the virus with high specificity in laboratories without PCR equipment, or a qualitative immunochromatographic test developed to be used on-site, which allowed rapid and specific detection in the field (e.g., on farms).^52^ Finally, serologic analyses to detect antibodies against RHDV2 were performed with a competitive ELISA already developed for RHDV strains and later used in epidemiologic surveys of RHDV2.^28,103,169^

### Africa

Detections of RHDV2 were first reported in 2015 in 3 locations in Africa and close-by islands. The virus was detected in domestic rabbits in Tunisia; in domestic and wild European rabbits in Tenerife, an island of the Spanish Canary Islands archipelago; and in domestic rabbits in the Republic of Benin.^22,121,144,176^ In Tunisia and the Canary Islands, the virus was identified during routine surveillance of annual RHD outbreaks, and the specific date of introduction was unknown. Phylogenetic analysis showed that the Tunisian viruses originated from a single introduction of European strains, most consistent with strains from Italy, which was suspected to have occurred between 2012 and 2014,^144^ whereas viruses from Tenerife had the highest nucleotide identity with Portuguese strains.^121^

Subsequently, reports from Ivory Coast suggested that RHDV2 was present in that country in 2016, because both rabbits and hares were affected, but the virus type was not reported to be RHDV2 until 2021.^114,187,188^ In 2017, RHDV2 was first detected in Morocco in samples obtained from wild rabbits, and the strains identified were GI.1bP-GI.2 recombinants, as found in the Iberian Peninsula and in the Azores and Madeira archipelagos^114^; RHDV2 circulated widely in Morocco, at least up until 2021.^153^ Subsequently, in 2018, the virus was detected in Egypt during regular surveillance for RHD outbreaks, so the date of the first introduction of the virus in that country is also unknown.^67^ An August 2023 study reported that RHDV2 was circulating in the North of Algeria since at least 2018^152^; the viruses were most similar to the GI.3P-GI.2 recombinant and the earlier detections clustered with Tunisian viruses, whereas the most recent (2020–2021) samples included in the analysis were more similar to some North American viruses.^152^ RHDV2 was first reported in commercial rabbit farms in Ghana in 2019,^12^ and in domestic rabbits in Senegal and Nigeria in 2020.^55,193^ Phylogenetic analysis of *vp60* and complete sequences obtained from microbial metagenomic sequencing techniques showed that Nigerian strains had high homology with RHDV2 viruses from the Netherlands and Germany, suggesting possible virus importation.^54,55,79^ A subsequent study confirmed that RHDV2 had been introduced into Africa several times, with independent incursions into, at least, Tunisia, Nigeria, and Morocco, and was most likely of European origin.^22^ RHDV2 was detected in South Africa in November 2022 and, per reports to WOAH, has affected domestic rabbits and native wild rabbits and hares.^195^ As multiple RHDV2 detections occurred in western and northern African coastal countries, it was suggested that the virus entered each country through legally or illegally imported rabbits or related products.^12,22,144^

### Oceania

Detections of RHDV2 in Oceania have been reported in Australia and New Zealand.^175,191^ The history of pathogenic lagoviruses in Australia is unique. Prior to the emergence of RHDV2, RHDV had circulated widely, mainly as a result of its use to control populations of invasive wild European rabbits. The Czech V351 (CAPM V-351) RHDV strain (a GI.1c virus) was released for this purpose in 1995 and was considered to be an ancestor of some circulating pathogenic lagoviruses.^65,100,116^ In 2017, the Korean K5 strain (a GI.1a virus) was also released as a biocontrol agent.^116^ An incursion with active circulation of a different RHDV genotype GI.1a virus occurred in 2014.^118^ Additionally, several variants of the nonpathogenic RCV (GI.4) were present in Australia prior to the release of RHDV.^147,159^

RHDV2 was first detected in a wild European rabbit in Australia in May 2015.^76,175^ Three dead wild rabbits were tested as part of surveillance of RHD outbreaks prior to the release of the K5/GI.1a in the Canberra region. While field RHDV was found in 2 carcasses, 1 animal tested positive for RHDV2. Subsequent phylogenetic analysis revealed that the virus was a GI.1bP-GI.2 recombinant.^76,116^ This virus was similar to some viruses previously circulating in mainland Portugal and the Azores.^10,113^ RHDV2 was also detected in Tasmania in 2016.^116^ Retrospective serologic studies showed that RHDV2 had been circulating in Australia before its first detection, at least since spring 2014,^146^ which was also supported by studies on the evolutionary history of Australian viruses and further serologic studies.^116,138,158^ RHDV2 had a significant impact on the wild rabbit populations between 2014 and 2018, with average population declines of up to 60% and more pronounced reductions in southern and western Australia.^146^ Interestingly, only 18 months after its first confirmed detection, RHDV2 had replaced the circulating endemic RHDV viruses in Australia, and was also detected in domestic rabbits and European brown hares.^78,116^ Cases of RHDV2 infection continue to be detected in Australia; seroprevalence remained high until at least 2022, and it is thought to be the virus currently having the greatest impact on wild populations.^145^ However, a low percentage of RHDV-seropositive animals are still being detected, suggesting co-circulation to some extent and ruling out total extinction of RHDV.^145,146^ In fact, the previous occurrence of multiple pathogenic and nonpathogenic lagoviruses in Australia has shaped the dynamics of RHDV2, fostering the occurrence of multiple recombinants,^117^ and at least 7 different lagoviruses are believed to be circulating: RHDV genotype GI.1, RCV genotype GI.4, RHDV2 genotype GI.1bP-GI.2, RHDV-K5 genotype GI.1a, RHDV genotype GI.4eP-GI.1a, RHDV2 genotype GI.4cP-GI.2, and RHDV2 genotype GI.4eP-GI.2.^138^

The use of different ELISA strategies has therefore been fundamental in shaping the Australian lagovirus landscape even before the emergence of RHDV2.^145,147^ However, various PCR approaches are the current method of choice and the test by which the occurrence of RHDV2 was initially confirmed in Australia.^76^ A multiplex RT-PCR that was able to differentiate among all of the pathogenic lagoviruses circulating in Australia at the time was developed.^77^ The assay was even able to identify mixed infections in individual animals. In addition, a SYBR green–based RT-qPCR was optimized to detect and quantify lagovirus genetic material in a single PCR reaction.^77^ Monolayer cell cultures derived from European rabbit and European brown hare hepatobiliary organoids support the replication of several Australian RHDV2 (both rabbit and hare organoids) and RHDV (rabbit organoids only)^95^; this provides a useful alternative to traditional viral culture systems, which have proven very challenging for lagoviruses.

The situation in New Zealand was somewhat similar to that in Australia. Prior to the RHDV2 incursion, the biocontrol agent CAPM V-351 RHDV was detected in 1997, possibly as a result of an illegal introduction by farmers,^135^ and further releases were made in the following years. The K5 RHDV strain was also released in 2018 for a similar purpose. The nonpathogenic RCV (GI.4) was also present in New Zealand before 1997.^131^ RHDV2 was first detected in a wild rabbit collected from the South Island in May 2018, but analysis of samples from rabbits collected the previous year were also positive.^124,191^

### Asia

The first reports of RHDV2 occurrence in Asia came from Israel in 2018.^184^ The same country reported cases to WOAH in 2020 and 2021.^185^ The only publicly available reports from Israel referred to detections in domestic rabbits. Reports of RHDV2 occurrence in East Asia appeared in 2019–2020, including detections in Japan and China.^96,179^ Maritime Southeast Asia has also been affected, with detections in Singapore in 2020.^194^

Since 2019, RHDV2 has been detected in several prefectures in Japan.^71,96,162,165^ In May 2019, all 10 rabbits in an exhibition facility from Ehime Prefecture died with gross and microscopic lesions compatible with RHD; viral particles were visualized by electron microscopy and the diagnosis was confirmed by RT-PCR followed by partial *vp60* sequencing^96^; this was the first documented case of a pathogenic lagovirus in Japan in 17 y. At least one other case of RHDV2 was detected in Ibaraki Prefecture in 2019.^162^ Shortly afterward, in 2020, an outbreak with a mortality rate of 55% occurred in a colony of domestic European rabbits in a zoologic garden in Chiba Prefecture.^71^ Similarly, in May 2020, 11 of 15 domestic rabbits from an exhibition facility in Tochigi Prefecture died from RHD caused by RHDV2.^165^ Other prefectures were likely affected in 2020, but the viral type was not determined.^162^ Whole-genome sequencing (WGS) with recombination and phylogenetic analyses of several Japanese detections in 2019–2020 revealed that at least 2 different RHDV2 recombinants, GI.3P-GI.2 and GI.1bP-GI.2, were circulating, and were more similar to viruses found in Germany and Australia in 2017, respectively, suggesting that the Japanese cases were associated with different foreign incursions.^162^

In April–May 2020, large outbreaks of RHD due to RHDV2 were reported in farmed rabbits in Sichuan Province, China and later spread to other provinces.^44,89,108^ These were the first reports of RHDV2 detection in the country. Mortality was high, ranging from ~43% to >70%, and affected also young individuals and rabbits that had been vaccinated against RHDV. The Sichuan viruses were most closely related to detections from the Netherlands in 2016 and Germany in 2017, and were GI.1aP-GI.2 recombinants.^44,108,143^ Based on the phylogenetic similarities with these European strains and the reliance of the Chinese rabbit industry on imports from European countries, it was suggested that some of these introductions into China may have originated from rabbit batches or imported semen purchased in Europe.^44,108,143^ A 2023 study reported that a novel recombinant, distinct from the original Chinese strain, was detected between 2020 and 2021 in at least 3 different provinces (Sichuan, Jiangsu, Guangxi) separated by 1,000–1,500 km.^88^ The novel recombinant had structural genes of the RHDV2 strain first detected in China and a nonstructural component of an unclassified lagovirus genotype similar to a virus that had been identified in healthy wild rabbits in the country.^81,88^ This recombinant induced lower mortality and had a longer replication time than the RHDV2 genotype GI.1aP-GI.2 detected in some of the first Sichuan cases.^88^

In September 2020, Singapore reported their first documented cases of RHDV2.^163^ All cases occurred in domestic pet rabbits and involved at least 3 households from an urban area; 8 of 11 exposed rabbits died. Rabbits from the different households had had some degree of contact with each other, and intra- and inter-household transmission, including contact at a veterinary clinic, were highlighted as important modes of dissemination in this outbreak.^109,163^ The source of the outbreak could not be determined, and introduction via infected rabbits or contaminated feed was initially considered unlikely.^109^ No further cases were reported that year, and the outbreak was declared resolved in December 2020.^109^ Subsequent WGS and recombination and phylogenetic analyses revealed that the Singapore 2020 virus was a GI.4cP-GI.2 recombinant highly homologous (~99.2%) to certain Australian viruses circulating in rabbit and hare populations since 2017^99,117^; therefore, it was suggested that the origin of the Singapore 2020 cases might have been an introduction of a virus that originated in Australia, but the means by which this occurred were unknown.^99^

### The Americas

As of September 2023, RHDV2 detections in the Americas have been limited to North America, specifically to Canada,^177^ the United States,^196^ Mexico,^190^ and Cuba.^181^ Interestingly, no detections have been reported from South America. This region remains as one of the largest officially unaffected areas in the world, and continued surveillance is warranted in the current context of rapid viral spread.

RHDV2 was first detected in North America in August 2016 in a 4-mo-old rabbit from a hobby farm in Mont-Joli, Quebec, Canada; the farm had received rabbits from another farm that was also presumably affected by RHD.^13,177^ Mortality in the affected premises was 72–89%, and several surviving rabbits were seropositive by ELISA. Sequences from this strain of RHDV2 were most similar to a virus collected in 2011 in Navarra, Spain.^13^ Following this outbreak, RHDV2 was not detected again in Canada until 2018.^178^ In December 2017, a significant mortality event was reported in feral rabbits from a private property south of Nanaimo, British Columbia but was not further investigated; high mortality was then reported in a population of feral domestic rabbits from the Vancouver Island University campus in early 2018.^13^ Additional cases in domestic rabbits were reported in mainland British Columbia through the summer of 2018.^13,85^ The strain of RHDV2 detected in British Columbia in 2018 shares only ~93% sequence homology with the strain detected in Quebec in 2016 and is thought to represent a separate incursion.^13^ In 2019, additional detections were reported in the region; of note, there was one case involving pet rabbits in a downtown Vancouver apartment that shares only ~97% identity at the nucleotide level with the 2018 British Columbia index case and is thought to represent a separate incursion into the region.^13^ Between 2020–2023, RHDV2 was detected several times in feral and captive domestic rabbits in other Canadian provinces in addition to British Columbia and Quebec, including Ontario and Alberta.^72^

RHDV2 was first detected in the United States in 2018, affecting pet rabbits in Ohio,^196^ and, shortly thereafter, in feral domestic rabbits in Washington State in 2019.^172^ The sequences of the viruses from Ohio and Washington State were both similar to a strain detected in British Columbia in 2018.^166^ In March 2020, RHDV2 was reported in domestic rabbits from a veterinary clinic in New York City, likely representing an introduction different from the Ohio and Washington State cases.^134,166^

In March 2020, RHDV2 was detected in domestic rabbits in New Mexico.^197^ Since then, the virus has spread extensively throughout the southwestern United States and later to other areas of the country; RHDV2 is now considered to be endemic in most of the western United States, with sporadic detections reported in some eastern states.^197^ The outbreak in the southwestern United States represents a new milestone in the global spread of RHDV2 as it is the first time that natural cases of RHD were reported in *Leporidae* species native to North America, including the black-tailed jackrabbit (*Lepus californicus*), antelope jackrabbit (*Lepus alleni*), desert cottontail (*Sylvilagus audubonii*), eastern cottontail, mountain cottontail (*Sylvilagus nuttallii*), western brush rabbit (*Sylvilagus bachmani*), riparian brush rabbit (*Sylvilagus bachmani riparius*), and pygmy rabbit (*Brachylagus idahoensis*); detections in domestic and feral domestic European rabbits occurred in parallel.^17,47,101,166,197^ Due to the high diversity of lagomorph species in the United States and the fact that reliable species identification is not always possible, it is likely that other species have been affected.

The first public complete RHDV2 sequences from the United States were made available in a 2020 study^134^; 3 sequences collected in Texas, New Mexico, and Arizona between March and April 2020, at the beginning of the southwest outbreak, had high nucleotide identity (almost 99.5%) and formed a cluster distinct from the New York 2020 detections. Sequences from California collected in late 2020 and early 2021 were highly homologous (99–99.4%) to the southwestern sequences reported in the aforementioned 2020 study.^18,134^ No comprehensive recombination studies have yet been published, but analyses of the topology of the phylogenetic trees of the structural and nonstructural genes of the 2020–2021 California sequences suggest that they were GI.3P-GI.2 recombinants.^18^ Because the virus has circulated widely and no detailed phylogenetic and recombination studies have been published yet, it is possible that other recombinants are present in the country. Of the available sequences from North America reported before 2020, the aforementioned southwestern U.S. sequences were more similar to one of the 2018 detections in British Columbia^13,18^; however, at this stage, it cannot be excluded that some of the cases of the southwestern outbreak and further detections in other parts of the country represent different incursions. In fact, RHDV2 detections continue to date, and as of 2023 Sep 30, the virus has been reported in 29 states, including Hawaii (USDA-APHIS RHDV2 outbreak map: https://www.aphis.usda.gov/aphis/maps/animal-health/rhd [cited Oct 2023]). Kohl and collaborators of the “RHD Awareness Team” from the University of Georgia are performing an up-to-date follow-up of the cases in the United States, compiling information from the USDA-APHIS and other sources, and the results can be viewed at https://rhdv2.org/ (cited Oct 2023).

In April 2020, almost at the same time as the first cases of the southwestern U.S. outbreak were being notified, detections were also reported in several northern states of Mexico.^190^ The virus has been reported in wild and domestic *Leporidae* species from at least 19 states, and is considered to be endemic in Mexico as of 2021; a sequence from Chihuahua available in GenBank (MT982431.1) clusters with the 2020–2021 southwestern U.S. sequences.

Several detections of RHDV2 from Cuba have been reported to WOAH since 2021, mostly in farmed rabbits.^181^ Further details of the spread and sequences are not available, but per the mentioned reports, multiple premises in different cities have been affected with up to thousands of animals being lost due to viral infection or culling.

During the recent outbreaks in the Americas, some novel detection approaches have been attempted. RT-qPCR from dried blood filter paper and ear punch samples obtained from cottontail rabbits (*Sylvilagus* spp.) and jackrabbits (*Lepus* spp.) had 100% sensitivity and specificity compared to liver at initial sampling and remained high after 6 wk, representing good alternative sample types to liver for RHDV2 surveillance in wild *Leporidae* species.^93^ Another study compared a post-freezing and thawing magnetic-bead RNA extraction method with a single-tube rapid swab extraction method after a second freeze–thaw cycle, and demonstrated an ~1 log_10_ reduction in sensitivity with the latter.^86^ In a California study, sensitivity and specificity of RT-qPCR from rectal swabs obtained from a pool of domestic rabbits and native North American leporids were 88% and 100%, respectively, compared with liver, suggesting that this sampling method can be used as a strategy to screen carcasses for viral RNA.^16^ A pan-lagovirus immunohistochemistry (IHC) test using antibodies obtained from the WOAH RHD Reference Laboratory (Brecia, Italy; provided by Drs. A. Lavazza and L. Capucci) has been used successfully as a complementary detection tool in domestic rabbits and wild *Leporidae* species native to North America.^16,18^ In addition, RNAscope (ACDBio)-based in situ hybridization has proven very effective in localizing viral nucleic acid in different cell types.^137^

## Affected animal species

### Oryctolagus

The first detections of RHDV2 were reported in domestic and wild European rabbits.^103,105^ The virus has circulated widely in this species, particularly in Europe and Oceania, and the vast majority of the experimental infections have been carried out using European rabbits. It is, therefore, the species in which the various aspects of the disease have been better characterized. As data for other species are much scarcer, disease features in European rabbits are often extrapolated to other *Leporidae* species, although differences are likely to occur.

Mortality associated with RHDV2 in European rabbits varies among different reports of natural disease, and is generally 10–90%, or occasionally up to 100% in small, naïve domestic populations.^18,75^ In wild populations, high mortality has been reported, particularly when the virus first occurs in a geographic area, and this has been one of the reasons the European rabbit has been listed as “endangered” in its natural range.^94^ Case fatality in experimental settings is also variable, 0–100%,^75,88,164^ and 42.6–48.2% in experimental settings with the highest number of animals used (*n* = 94^30^ and *n* = 110^106^). The time from inoculation to death or euthanasia was 18 h to 9 d in the reports of experimental infections,^75,88,164^ with a mean time to death or euthanasia after inoculation of ~53 h in one of the experiments with the highest number of inoculated animals (*n* = 94^30^). Some experimental studies did not find differences in mortality rates and clinical signs between adult rabbits and kittens.^30,75^ However, in a 2022 experiment,^164^ shorter clinical courses and higher viral loads were observed in 4-wk-old kittens compared to adults and subadults. Significantly shorter survival time and time of onset of pyrexia were observed in 5-wk-old kittens compared with 11-wk-old rabbits in a different study.^75^ Overall, differences in disease course and mortality may be due to genetic differences in the RHDV2 viruses involved, route and dose of infection, previous immunity to lagovirus infection (including other pathogenic and nonpathogenic lagoviruses, and vaccination), and other epidemiologic parameters.^32,75^

**Figures 4–9.**
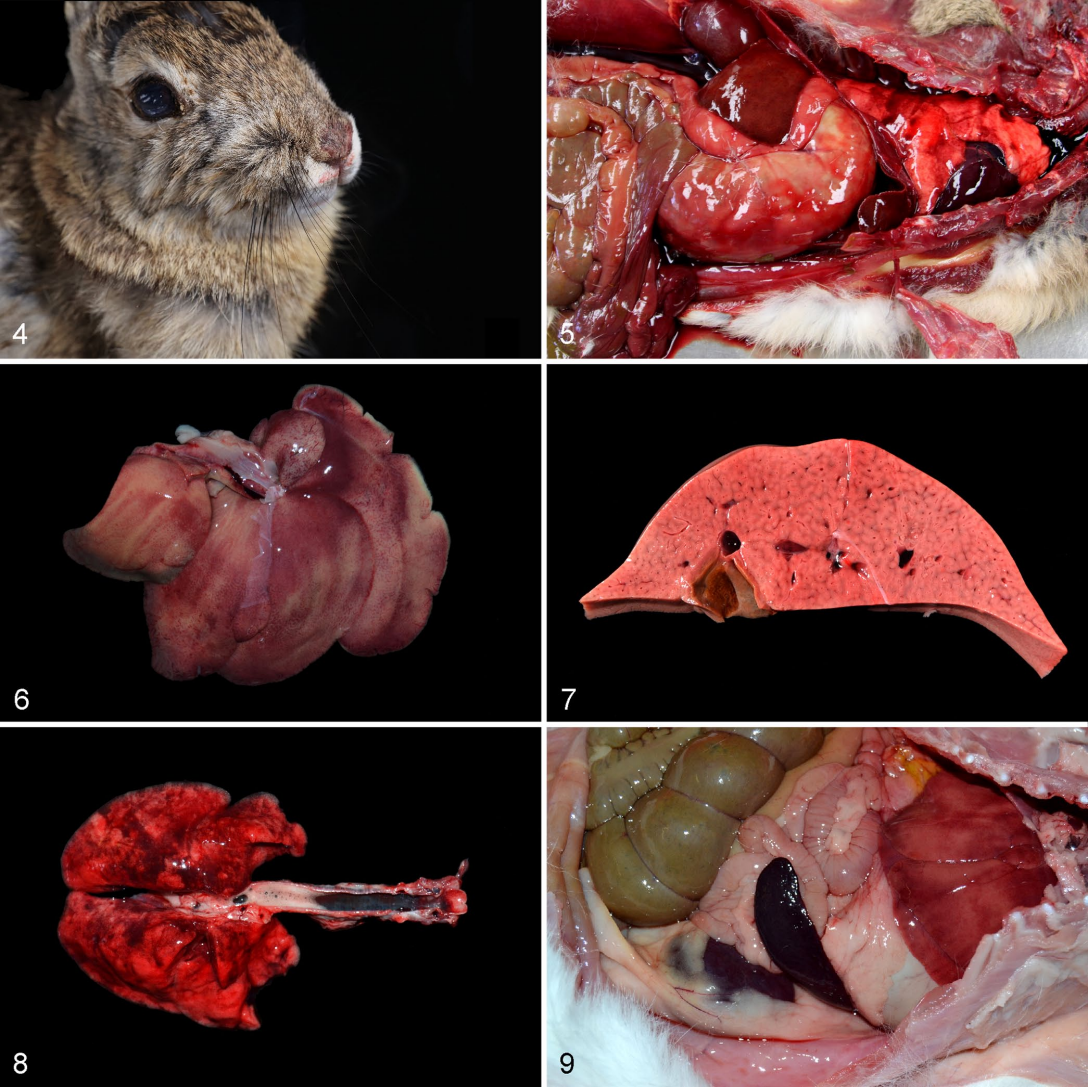
Gross lesions of rabbit hemorrhagic disease virus 2 infection. **Figure 4.** Desert cottontail rabbit with blood around the nares. **Figure 5.** Black-tailed jackrabbit with pulmonary and serosal congestion and hemorrhages, and blood in abdominal cavity. Photo courtesy of Dr. Akinyi Nyaoke, UC Davis. **Figures 6–9.** Domestic rabbits. **Figure 6.** Diffuse hepatic pallor and reddening of the capsule with enhanced reticular pattern. **Figure 7.** Cut section of liver with pallor and reticular pattern. **Figure 8.** Pulmonary hemorrhages and congestion. Tracheal congestion and foam in the lumen. **Figure 9.** Marked splenomegaly and mild hepatic pallor.

The clinical signs and pathology (Table 1) of RHDV2 infection in the European rabbit have been described in some detail in both natural and experimental cases.^9,30,49,75,80,128,141,172^ Major clinical signs include sudden death, fever, collapse, lethargy, seizures, icterus, oral bleeding, dyspnea, hypothermia, bradycardia, poor blood clotting, epistaxis, apathy, depression, anorexia, ataxia, and circling. Elevations in serum transaminases and alkaline phosphatase activities and bilirubin have been noted in pet rabbits^24,141,163^; however, one report mentioned significant decreases in aspartate aminotransferase and alanine aminotransferase activities in 2 pet rabbits, perhaps related to the short half-life of these enzymes in rabbits.^24^ Peracute, acute, subacute, and chronic clinical presentations are described in individual animals. Sudden death without additional signs was frequently recorded in a survey of pet rabbits,^80^ and it is thought to be the most common outcome in peracute disease. Subacute-to-chronic clinical courses were relatively common in the first reports of RHDV2.^103^ Epistaxis or serosanguineous nasal discharge (Fig. 4) is considered a classic clinical sign of RHD; however, a large survey of commercially farmed rabbits found no clear association between serosanguineous nasal discharge and RHDV2 infection,^150^ and therefore the absence of this finding should not preclude a presumptive diagnosis of RHD in cases of mass mortality.

**Table 1.** Gross and microscopic lesions of rabbit hemorrhagic disease virus 2 in European rabbits (*Oryctolagus cuniculus*).

Organ	Gross lesion	Microscopic lesion
Liver	Hepatomegaly, pallor, hemorrhages, marked acinar/lobular pattern, tan subcapsular foci	Necrosis (periportal, massive, or random); hepatocyte individualization, hypereosinophilia, pyknosis, karyorrhexis, karyolysis; vacuolar degeneration (lipid type); cholangitis, portal fibroplasia, proliferation of bile ducts (subacute-to-chronic forms); hepatocyte mineralization (subacute-to-chronic forms); multinucleated hepatocytes (subacute-to-chronic forms)
Lung	Congestion, hemorrhages (petechiae to more widespread distribution), foam and serosanguineous fluid in airways	Intraalveolar and perivascular hemorrhages, alveolar edema, microthrombi in septal capillaries and lobular small to medium-sized vessels
Spleen	Splenomegaly, friability, dark-red to black discoloration	Hyaline necrosis of red pulp, congestion and hemorrhage, lymphocytolysis in white pulp, microthrombi in central arterioles
Kidney	Pallor to reddening, petechiae	Interstitial, cortical, and medullary hemorrhages; microthrombi in glomerular and interstitial capillaries; tubular epithelial degeneration and necrosis; protein or hemoglobin casts
Heart	Petechial-to-paintbrush hemorrhages in epicardium and endocardium	Myocardial hemorrhages, cardiomyocyte degeneration and necrosis
Trachea	Dark-red mucosal discoloration, abundant foam	Markedly congested submucosal and lamina propria capillaries, mucosal mineralization
Stomach and intestines	Gastric and cecal dilation and stasis	Superficial and crypt epithelium necrosis, capillary microthrombi and hemorrhages in lamina propria capillaries
Thymus	Petechiae	Lymphocytolysis
Brain	–	Microthrombosis and rarefaction of adjacent neuropil, mild lymphoplasmacytic perivascular meningoencephalitis
Reproductive tract	Serosal hemorrhages in the uterus	–

Adapted from references Abade dos Santos^1^ and Henning^82^. – = no significant lesions.

On gross postmortem examination (Table 1; Figs. 4–9), a lobular or reticular pattern in the liver is frequently, albeit not always, observed, and overall enlargement, pallor, and friability usually affect this organ (Figs. 6, 7, 9). There is usually congestion and hemorrhage in several organs, being particularly severe in the liver and lungs (Figs. 5, 8). Dark-red discoloration of the tracheal mucosa has been noted as a distinctive gross change (Fig. 8). Serosanguineous fluid or blood in body cavities has also been described as a distinctive feature (Fig. 5). Less commonly, the spleen is enlarged (Fig. 9), and icterus is occasionally present. Importantly, gross lesions may be minimal to absent in some animals with RHD caused by RHDV2,^9,80^ and in wild and feral domestic rabbits, gross and microscopic lesions are usually obscured by postmortem decomposition or freeze–thaw artifacts.^172^

**Figures 10–13.**
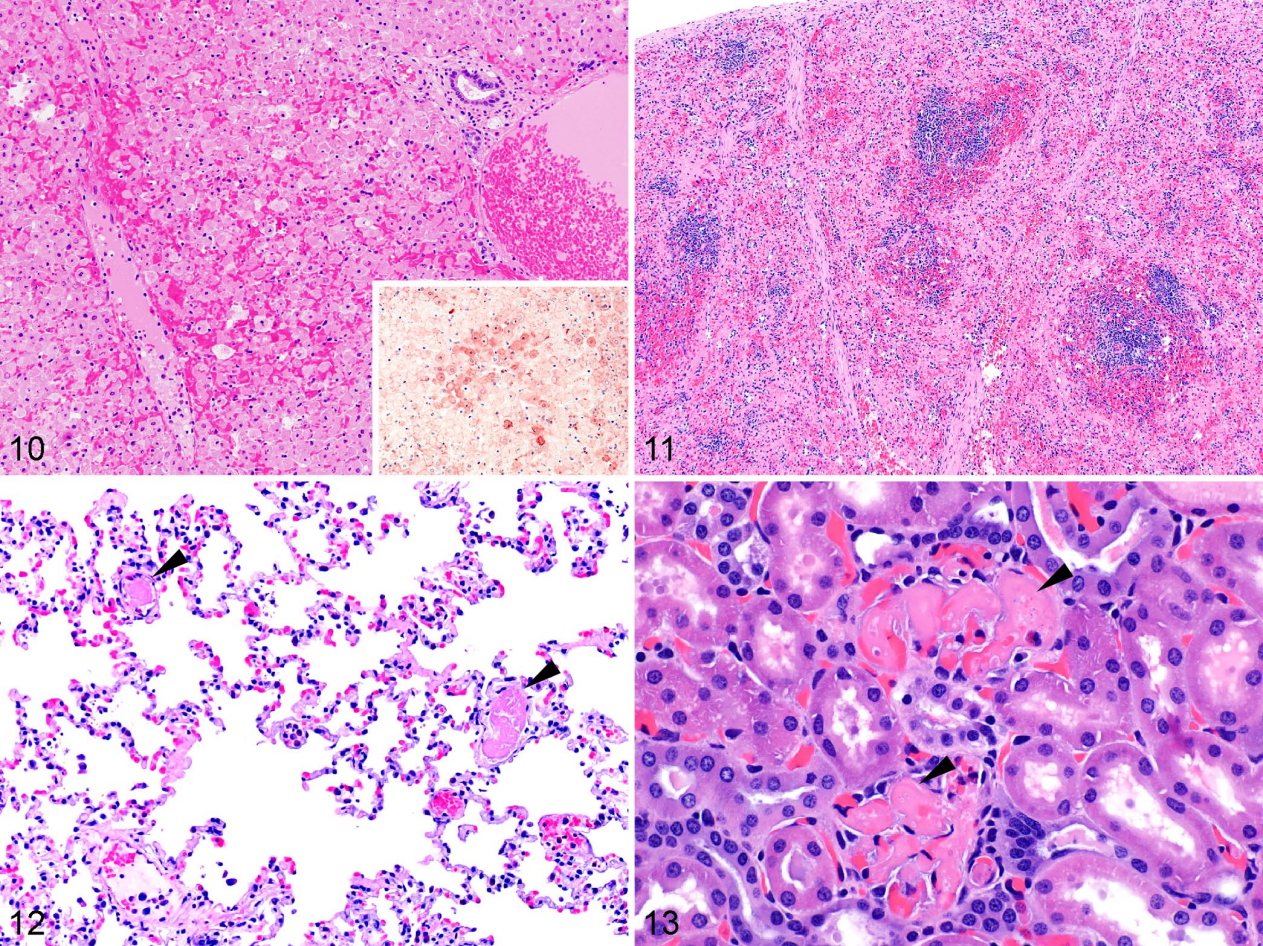
Microscopic lesions of rabbit hemorrhagic disease virus 2 infection. H&E. **Figure 10.** Liver from a riparian brush rabbit, with hepatic necrosis, disorganization of hepatocyte cords, hypereosinophilia, pyknosis, karyorrhexis, and hemorrhage. Inset: positive pan-lagovirus immunohistochemistry. Case kindly donated by Dr Peter Chu, UC Davis. **Figures 11–13.** Domestic rabbits. **Figure 11.** Lymphoid depletion of follicles in the white pulp of the spleen, and red pulp necrosis with seams of hyaline eosinophilic material. **Figure 12.** Fibrin thrombi in 2 pulmonary vessels (arrowheads). **Figure 13.** Fibrin thrombi (arrowheads) in glomerular capillaries.

Histologically (Table 1; Figs. 10–13), hepatic necrosis with hepatocellular dissociation and apoptosis, and a periportal-to-massive distribution, is the hallmark and consistent microscopic lesion (Fig. 10). This lesion usually has diagnostic value in cases with limited gross changes. However, hepatic necrosis with random rather than periportal distribution is occasionally observed,^172^ which suggests that the pattern of hepatic necrosis should not always be considered as an unequivocally distinctive feature. Preparing overnight histologic sections may be effective in establishing a presumptive diagnosis of RHD while awaiting confirmatory PCR results (J. Asin, E.E. Henderson, and F.A. Uzal, personal observations). Hepatic necrosis is sometimes associated with congestion, hemorrhage, and sinusoidal thrombosis; mild-to-marked infiltration of heterophils and macrophages is frequently seen. The spleen may have deposition of hyaline eosinophilic material in the red pulp, marked hemorrhage and congestion, and lymphocytolysis in the white pulp (Fig. 11). Pulmonary congestion, edema, and hemorrhage are observed in most cases, usually with microvascular thrombosis (Fig. 12). Microthrombi in glomerular capillaries (Fig. 13) and acute tubular injury are occasionally seen in the kidneys. Microthrombosis can be observed in virtually any organ and is the result of disseminated intravascular coagulation and perhaps viral infection of endothelial cells (Table 1).^80,137^

### Lepus

RHDV2 has been detected in a number of hare and jackrabbit species (Table 2).^25,26,34,104,142,166,168,195^ Most cases of RHDV2-associated mortality in hares coincided with concurrent outbreaks in rabbits, suggesting the role of rabbits as the primary source of infection to initiate outbreaks in hares. However, with the exception of some outbreaks such as the one that occurred in Cape hares in Sardinia, Italy,^142^ in which a significant number of hares were affected, mortality is limited in the available reports and generally lower than in European rabbits,^34^ suggesting the occurrence of spillover events from rabbits to hares favored by the high infection pressure during outbreaks in the former, but causing only sporadic infections in the latter.^169^ Transmission between infected hares has been postulated but not definitively demonstrated experimentally.^104,129^ Interestingly, viruses collected from hares can also have high genetic variability. For instance, 2 RHDV2 genotype GII.1P-GI.2 recombinants were collected from European brown hares in Germany in 2014 and 2019,^160^ and a GI.1bP-GI.2 recombinant was detected in the same species in Australia in 2016.^78,116^

**Table 2.** Detections of rabbit hemorrhagic disease virus 2 in *Leporidae* species other than European rabbit (*Oryctolagus cuniculus*).

Common name	Scientific name	Country (island)	Reference
Antelope jackrabbit	*Lepus alleni*	USA	^166,197^
Black-tailed jackrabbit	*Lepus californicus*	USA, Mexico	^17,101,166,197^
Cape hare	*Lepus capensis*	South Africa	^195^
Desert cottontail	*Sylvilagus audubonii*	USA, Mexico	^17,101,166,197^
Eastern cottontail	*Sylvilagus floridanus*	USA	^125,166,197^
European brown hare	*Lepus europaeus*	Italy, Spain, France, Australia, England, Scotland, Germany	^21,68,78,104,148,169^
Iberian hare	*Lepus granatensis*	Spain	^168^
Irish hare	*Lepus timidus hibernicus*	Ireland	^26,97^
Italian hare	*Lepus corsicanus*	Italy (Sicily)	^34^
Mountain cottontail	*Sylvilagus nuttallii*	USA	^166,197^
Mountain hare	*Lepus timidus*	Sweden (Hallands Väderö), Germany	^25,129^
Pygmy rabbit	*Brachylagus idahoensis*	USA	^47^
Red rock rabbit	*Pronolagus* spp.	South Africa	^195^
Riparian brush rabbit	*Sylvilagus bachmani riparius*	USA	^27^
Sardinian Cape hare	*Lepus capensis mediterraneus*	Italy (Sardinia)	^142^
Scrub hare	*Lepus saxatilis*	South Africa	^195^
Western brush rabbit	*Sylvilagus bachmani*	USA	^16^

Descriptions of lesions in hares and jackrabbits are limited and often based on single case reports of natural disease. Nevertheless, gross findings in hares and jackrabbits appear to be similar to those described in European rabbits with RHD and also in European brown hares with EBHS (Fig. 5).^119^ Friable discolored livers with a prominent reticular pattern, congestion of viscera (particularly of the tracheal mucosa), splenomegaly, disseminated hemorrhages, and pulmonary edema have been described.^17,25,26,34,97,101,129,142,168,169^ Histologic findings are typically hepatic necrosis and apoptosis, usually with a massive distribution, hepatocellular fatty degeneration, and marked congestion and hemorrhage.^129,142,169^ Periportal-to-midzonal necrosis has been described in an Iberian hare.^168^ Lesions in black-tailed jackrabbits in North America are similar to other members of the genus *Lepus* elsewhere, and some cases have had periportal-to-midzonal hepatocellular dissociation and necrosis or apoptosis.^17,101^ Hepatocyte mineralization may be a more common feature in some members of this genus compared to other *Leporidae* species, as it has been described in certain species such as black-tailed jackrabbits and mountain hares.^101,129^ Interestingly, the latter parallels a comparative description of EBHS in European brown hares and RHD caused by classic RHDV in European rabbits.^119^ Some lesions typically associated with RHD in European rabbits, such as microthrombosis, heterophilic inflammation associated with the areas of hepatic necrosis, or lymphoid depletion and other splenic changes, may not be as common in black-tailed jackrabbits.^101^ Viral antigen has been detected by IHC in several hare and jackrabbit species using different antibodies.^18,129,168^ In a study of California cases,^16^ liver viral loads were significantly higher in a group of 5 black-tailed jackrabbit carcasses compared to 55 domestic European rabbits (median RT-qPCR Ct of 11.5 [*Lepus*] vs. 13.0 [*Oryctolagus*]).

### Sylvilagus

The first detections of RHDV2 in rabbits of the genus *Sylvilagus* were reported in the southwestern United States in April–May 2020.^166^ Based on publicly available information, the virus has been detected in at least 5 species of the genus *Sylvilagus* (Table 2).^16–18,27,101,166,197^ Other rabbits of this genus native to the Americas are also likely to be susceptible to the virus, and although they may have been affected, data on these species are not yet publicly available. Interestingly, the virus has been detected widely in desert cottontails in the western United States, whereas detections in eastern cottontails in other parts of the country have been rare.^197^ However, according to publicly available records, researchers at the Plum Island Animal Disease Centre (NY, USA) demonstrated the susceptibility of the eastern cottontail to develop RHD after experimental inoculation with RHDV2.^125^ Interestingly, the eastern cottontail is not susceptible to experimental infection with classic RHDV,^125^ but develops an EBHS-like disease when inoculated with EBHSV.^102^

Descriptions of RHD lesions in rabbits of the genus *Sylvilagus* are limited, but the changes appear to be similar to those observed in European rabbits, with hepatic necrosis or apoptosis being the main diagnostic finding (Fig. 10).^18,101^ Three of 5 eastern cottontails inoculated orally with an RHDV2 genotype GI.1bP-GI.2 recombinant in the Plum Island study^125^ developed a disease consistent with RHD with pale friable liver, petechiae, and serosanguineous fluid in the abdomen grossly, and periportal hepatocellular degeneration and necrosis with heterophilic inflammation histologically. Interestingly, 1 of the 2 eastern cottontails that did not succumb to the disease developed a high antibody titer (1:2,560) by the end of the 21-d experimental period. A 2021 study provided a detailed description of natural lesions in 7 desert cottontails collected in the southwestern United States in April and May 2020.^101^ Similar to black-tailed jackrabbits, certain histologic lesions typically associated with RHD in European rabbits were not seen in these animals, including glomerular or pulmonary fibrin microthrombi; heterophilic inflammation associated with areas of hepatic necrosis; and splenic lymphoid depletion, lymphocytolysis, macrophage hyperplasia, or red pulp necrosis. Hepatic necrosis has been observed in cases of RHD in western brush rabbits and riparian brush rabbits in California (Fig. 10).^16^ Descriptions of lesions in mountain cottontails and other members of the genus *Sylvilagus* are not currently in the public domain, but are thought to be similar.

Viral antigen has been detected by IHC in the liver and other organs of eastern cottontails, desert cottontails, western brush rabbits, and riparian brush rabbits (Fig. 10).^16,18,125^ A mouse monoclonal antibody cocktail directed against several capsid epitopes (6G2, 3H6, 6D6) of pathogenic and nonpathogenic lagoviruses from the WOAH RHD Reference Laboratory (Brescia, Italy; provided by Drs. A. Lavazza and L. Capucci) was used in all cases.^40^ Antigen and antibody ELISAs from the WOAH reference laboratory were used in liver homogenates and serum, respectively, from experimentally infected eastern cottontails.^125^ In a study of California cases,^16^ liver viral loads were significantly higher in a pool of carcasses of rabbits of the genus *Sylvilagus* (12 desert cottontails, 1 western brush rabbit) compared to 55 domestic European rabbits (RT-qPCR median Ct values of 11.3 [*Sylvilagus*] vs. 13.0 [*Oryctolagus*]).

### Other lagomorph species

As the virus spreads around the globe, other lagomorph species restricted to specific geographic niches are being exposed. Mortality events were reported in the pygmy rabbit in Nevada (USA) in January–February 2022, and one rabbit tested positive by RT-qPCR.^47^ Gross findings in affected rabbits included pulmonary congestion and hemorrhage and mild hepatic discoloration, but histopathology was not performed. Early 2023 detection reports to the WOAH include mortality in populations of red rock rabbits (syn. red rock hares; *Pronolagus* spp.) in South Africa.^195^ To our knowledge, no further information is publicly available, but this would represent a very novel species jump within the family *Leporidae* (Fig. 1).

Detection in previously unaffected lagomorph species is one of the main concerns of the current rapid global spread, particularly from a conservation standpoint. Concerns focus on unique lagomorph species such as pikas (*Ochotona* spp.) or volcano rabbits (*Romerolagus diazi*), among others. There is no confirmed information about detections in these species, but the virus is present in the geographic areas where some of these unique animals live, and awareness is warranted.

### Non-lagomorph species

RHDV2 RNA has been sporadically detected in non-lagomorph species from at least 4 different orders (Table 3). These specimens included 1 Mediterranean pine vole (*Microtus duodecimcostatus*) collected in Spain in 2014^31^; 2 white-toothed shrews (*Crocidura russula*), also collected in Spain 1 y later^31^; and several specimens of Eurasian badgers (*Meles meles*) collected in Portugal between 2017 and 2020.^5^ In addition to the detection of viral RNA, the infectivity of the virus detected in the livers of the Mediterranean pine vole and the 2 white-toothed shrews was demonstrated by inoculation of laboratory New Zealand White rabbits (*O. cuniculus*) that died with RHD lesions.^31^ Furthermore, RHDV2 was detected in the feces of Tasmanian devils (*Sarcophilus harrisii*) and red foxes (*Vulpes vulpes*) collected between 2017 and 2019 in Australia.^36,45^

**Table 3.** Rabbit hemorrhagic disease virus 2 detections in non-lagomorph species.

Common name	Scientific name	Order	Year of collection	Biological material tested	Country, region	Method used for detection	No. of positive specimens	Ref.
Eurasian badger	*Meles meles*	*Carnivora*	2017–2020	Liver, spleen, lungs, etc.	Portugal, Santarém district	RT-qPCR^61^	10	^5^
Mediterranean pine vole	*Microtus duodecimcostatus*	*Rodentia*	2014	Liver	Spain, Zaragoza Province	Duplex RT-qPCR^30^	1	^31^
Red fox	*Vulpes vulpes*	*Carnivora*	2019	Feces	Australia	Metagenomics & transcriptomics	2	^36^
Tasmanian devil	*Sarcophilus harrisii*	*Dasyuromorphia*	2017	Feces	Australia, Tasmania	Metagenomics & transcriptomics	1	^45^
White-toothed shrew	*Crocidura russula*	*Eulipotyphla*	2015	Liver	Spain, Zaragoza Province	Duplex RT-qPCR^30^	2	^31^

## Discussion and conclusions

Since its first reported detections in Europe in 2010,^103^ the literature reviewed here suggests that RHDV2 has been more evolutionarily successful than classic RHDV strains. The detection of RHDV2 in a wider range of species is a significant improvement in its ability to survive, but nevertheless a serious concern, particularly from the standpoint of animal conservation and welfare.

RHDV2 continues its rapid global spread from its original wild and domestic rabbit host in Europe to several wild *Leporidae* species and previously unaffected geographic areas, mainly as a result of human intervention.^151^ The current near-global presence of this emerging virus also appears to be related to its many survival strategies, including the ability to infect kittens <2-wk-old^51^; the wider host range^18^; genetic recombination with limited cross-protective immunity between different genotypes^127^; apparent lower mortality,^106^ possibly with an associated higher percentage of chronically infected animals,^4^ which might be able to serve as virus reservoirs; prolonged disease progression^75^; and high environmental resistance, believed to be similar to RHDV.^83^

The apparent speed at which RHDV2 is evolving is likely related to 2 well-documented recombination sites at the *RdRp-vp60* and *p16*-*p23* gene boundaries.^6^ Significantly, several examples of recombination involving both pathogenic and nonpathogenic lagoviruses have been documented, even including recombination events at both of these genome breakpoints simultaneously.^6,155^ Importantly, there is very limited knowledge of nonpathogenic rabbit or hare caliciviruses circulating in wild *Leporidae* species in North America; therefore, recombinants involving the structural genes of RHDV2 (genotype GI.2) and viruses of unknown genotype may potentially occur.

The phylogenetic classification of lagoviruses is based on the *vp60* gene.^107^ WGS has been widely used to understand the phylodynamic and molecular epidemiology of the virus, and the use of sequence analysis is expected to continue as a targeted detection and surveillance tool. Sequencing of *vp60* and WGS allow for RHDV2 to be quickly identified, characterized, monitored, and potentially controlled through early detection and elimination. The 2020–2023 RHDV2 situation in North America underscores the importance of early detection and immediate intervention when dealing with a rapidly evolving RNA virus.^18^ The first known North American outbreak affecting domestic rabbits in Quebec, Canada in 2016 was traced to a Spanish parent virus,^13^ but this strain has not been detected since. In contrast, the strain detected in New Mexico (USA) in 2020 successfully spread to multiple states over 3 y, and has crossed the species barrier into native species in the genera *Sylvilagus*, *Lepus*, and *Brachylagus*.^18,47,166^

In this respect, the spread of the virus in North America since 2019–2020 represents an unprecedented situation. Prior to the incursion of RHDV2, there had been sporadic detections of classic RHDV and other pathogenic lagoviruses in Canada and the United States since at least 2000, but to our knowledge the virus never became endemic in those areas^14,23,35,66^; outbreaks of RHD caused by RHDV were also reported in domestic rabbits in Mexico in the late 1980s, but a successful control program led to viral eradication in the country.^74^ However, RHDV2 has become endemic in those geographic locations in a short time, which is perhaps related to the susceptibility of some of the wild *Leporidae* species native to the Americas combined with human intervention and the other epidemiologic advantages of this virus.^37^

Despite some subtleties, gross and microscopic lesions appear to be similar in every new *Leporidae* species in which the virus is detected.^101^ However, information on lesions in species of genera other than *Oryctolagus* is scarce, and detailed descriptions and further comparative studies are warranted as the virus is detected in new species. In general, gross lesions may occasionally be discrete and nonspecific and, therefore, of limited diagnostic value, but microscopic evidence of hepatic necrosis is undoubtedly the most important parameter to be sought in diagnostic pathology.^16^ Other organs that are useful to sample for histologic examination in RHD-suspect cases, at least in European rabbits, include the lungs, spleen, and kidneys.^128^ However, confirmatory diagnosis is most commonly made by the detection of viral RNA in tissue samples, ideally by RT-PCR or RT-qPCR from liver.^198^

As with any virus that is genetically capable of rapidly evolving and adapting to new host species, vigilance in early detection and targeted efforts to manage the spread will be critical in preventing outbreaks and minimizing further dissemination. RHDV2 models a typical RNA virus that exploits all evolutionary survival strategies. The lessons learned from this virus can be applied to other viruses and should be reviewed by scientists and policy makers before large-scale interventions are planned.
